# Standing Long-Leg Radiographs Underestimate Varus Alignment and Joint Line Obliquity Compared With Computed Tomography in Japanese Patients

**DOI:** 10.7759/cureus.114993

**Published:** 2026-08-22

**Authors:** Yoshika Hiratsuka, Akira Maeyama, Tetsuro Ishimatsu, Kotaro Miyazaki, Yoshiaki Hideshima, Takuaki Yamamoto

**Affiliations:** 1 Department of Orthopaedic Surgery, Fukuoka University, Fukuoka, JPN

**Keywords:** computed tomography, coronal plane alignment of the knee, joint line obliquity, long-leg radiograph, lower limb alignment, total knee arthroplasty

## Abstract

Objective

This study aimed to determine whether standing long-leg radiographs (LLR) underestimate the medial proximal tibial angle (MPTA) and lateral distal femoral angle (LDFA) compared with CT in a Japanese population, and to evaluate the resulting coronal plane alignment of the knee (CPAK) classification based on the arithmetic hip-knee-ankle angle (aHKA) and joint line obliquity (JLO).

Methods

This retrospective study included 90 patients (97 knees) who underwent robot-assisted total knee arthroplasty (TKA) between April 2024 and October 2025. Patients with previous surgery on the affected limb or post-fracture malunion were excluded. The MPTA and LDFA were measured using LLR and CT. The aHKA and JLO were calculated, and the CPAK classification was determined.

Results

There were significant differences between LLR and CT measurements for the MPTA, LDFA, aHKA, and JLO (p < 0.05). Compared with CT, LLR underestimated varus lower limb alignment and JLO. Although there was no significant difference between the two imaging modalities in the distribution of CPAK classifications, CT showed a higher proportion of types I-III and a lower proportion of types IV-V.

Conclusions

In a Japanese population, LLR underestimated the degree of varus lower limb alignment and JLO compared with CT. Although the distribution of CPAK classifications did not significantly differ between the two imaging modalities, CT showed greater varus alignment and lower JLO values than LLR, suggesting that it may be useful for evaluating knees near the boundaries between CPAK classification types.

## Introduction

Standing long-leg radiographs (LLRs) are commonly used to evaluate lower limb alignment in the preoperative planning of total knee arthroplasty (TKA) [1]. However, LLR measurements are influenced by factors such as limb position and weight-bearing conditions during imaging and may not accurately reflect a patient’s constitutional alignment [2,3]. In recent years, robot-assisted TKA using three-dimensional CT has become increasingly widespread, enabling a more objective and reproducible assessment of lower limb alignment [4,5]. In our institution, standing long-leg radiographs and preoperative CT scans are routinely used for alignment assessment in patients undergoing robot-assisted TKA. Although these imaging modalities differ in patient positioning and weight-bearing conditions, understanding the differences between them is clinically important because both are commonly used in preoperative planning. CT provides a morphology-based assessment that is less affected by limb position and weight-bearing conditions than standing radiographs.

Tarassoli et al. [1] reported that standing LLRs underestimate the degree of constitutional varus alignment and joint line obliquity (JLO) compared with CT. Furthermore, they suggested that discrepancies between imaging modalities may influence the determination of the coronal plane alignment of the knee (CPAK) classification, potentially leading to misclassification of alignment types [1]. The CPAK classification categorizes knee coronal alignment into nine phenotypes based on two parameters: the arithmetic hip-knee-ankle angle (aHKA) and JLO. These parameters are calculated based on the medial proximal tibial angle (MPTA) and lateral distal femoral angle (LDFA), which are considered reliable and reproducible measurements that are less affected by weight-bearing conditions [1]. Among patients with knee osteoarthritis, CPAK type I is reportedly the most common phenotype in Japanese populations (53.8%-63.4%) [6,7], whereas type II is the most prevalent phenotype in Western populations (32.2%) [8]. These findings indicate that coronal alignment characteristics differ between ethnic populations.

Previous studies have reported that varus alignment in Japanese patients is often tibial dominant [6,7]. However, distal femoral morphology may also influence overall lower limb alignment [9,10]. Considering these morphological characteristics, differences in measured angles between imaging modalities may not represent simple measurement error but may instead reflect population-specific anatomical features. Most studies comparing standing LLR and CT have been conducted in Western populations [11,12], and the influence of anatomical characteristics and constitutional alignment specific to Asian populations remains unclear. Asians have been reported to exhibit greater constitutional varus alignment than Western populations [13-15], and it remains uncertain whether similar discrepancies between imaging modalities occur in these populations.

As Japanese patients often demonstrate pronounced constitutional varus alignment, underestimation of varus alignment and JLO on LLRs may have greater clinical implications than in Western populations. Furthermore, in preoperative alignment assessment, the distribution of CPAK phenotypes may vary depending on the imaging modality used. However, this issue has not yet been investigated in Japanese patients. The aim of this study was to compare the MPTA and LDFA measured using standing LLR and CT in Japanese patients who underwent robot-assisted TKA at our institution and to evaluate the effects of these differences on aHKA, JLO, and CPAK classification.

## Materials and methods

Patients

**Figure 1:**
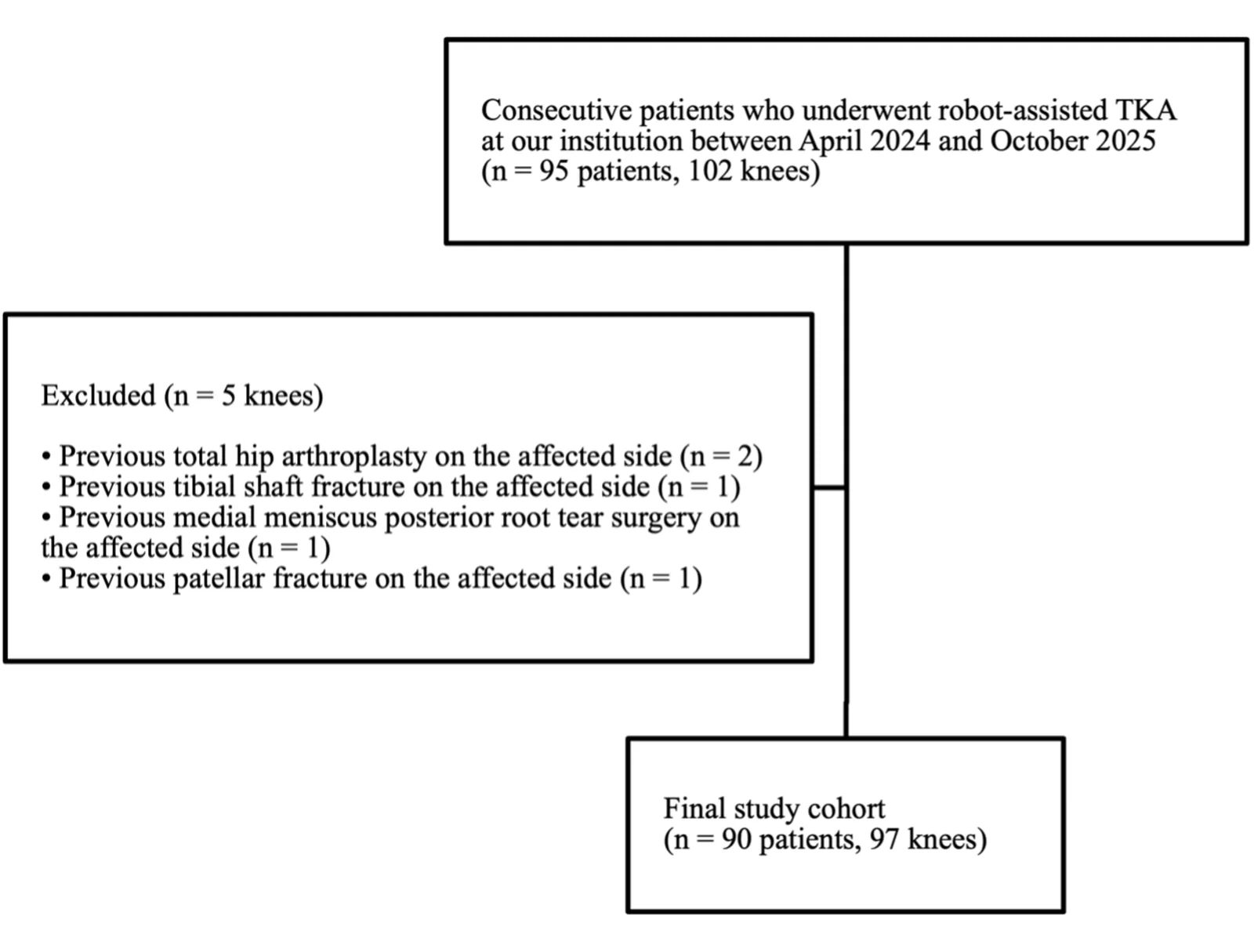
Patient enrolment flowchart TKA: total knee arthroplasty

This study was approved by the Ethics Committee of Fukuoka University Hospital (approval number: U25-08-018; approval date: August 25, 2025). We retrospectively reviewed patients who underwent robot-assisted TKA at our institution between April 2024 and October 2025. A total of 95 patients (102 knees) were initially identified, and the patient selection process is summarized in Figure 1. Patients with a history of previous surgery on the affected limb (four cases) and those with post-fracture malunion (one case) were excluded. Consequently, 90 patients (97 knees) were included in the final analysis.

Outcome measures

The primary outcome was the difference in MPTA and LDFA measurements between preoperative standing LLRs and CT. The secondary outcomes were the distribution of CPAK classification phenotypes based on aHKA and JLO, along with patient demographic factors, including age, sex, and BMI.

Imaging protocol and measurement methods

LLRs

**Figure 2:**
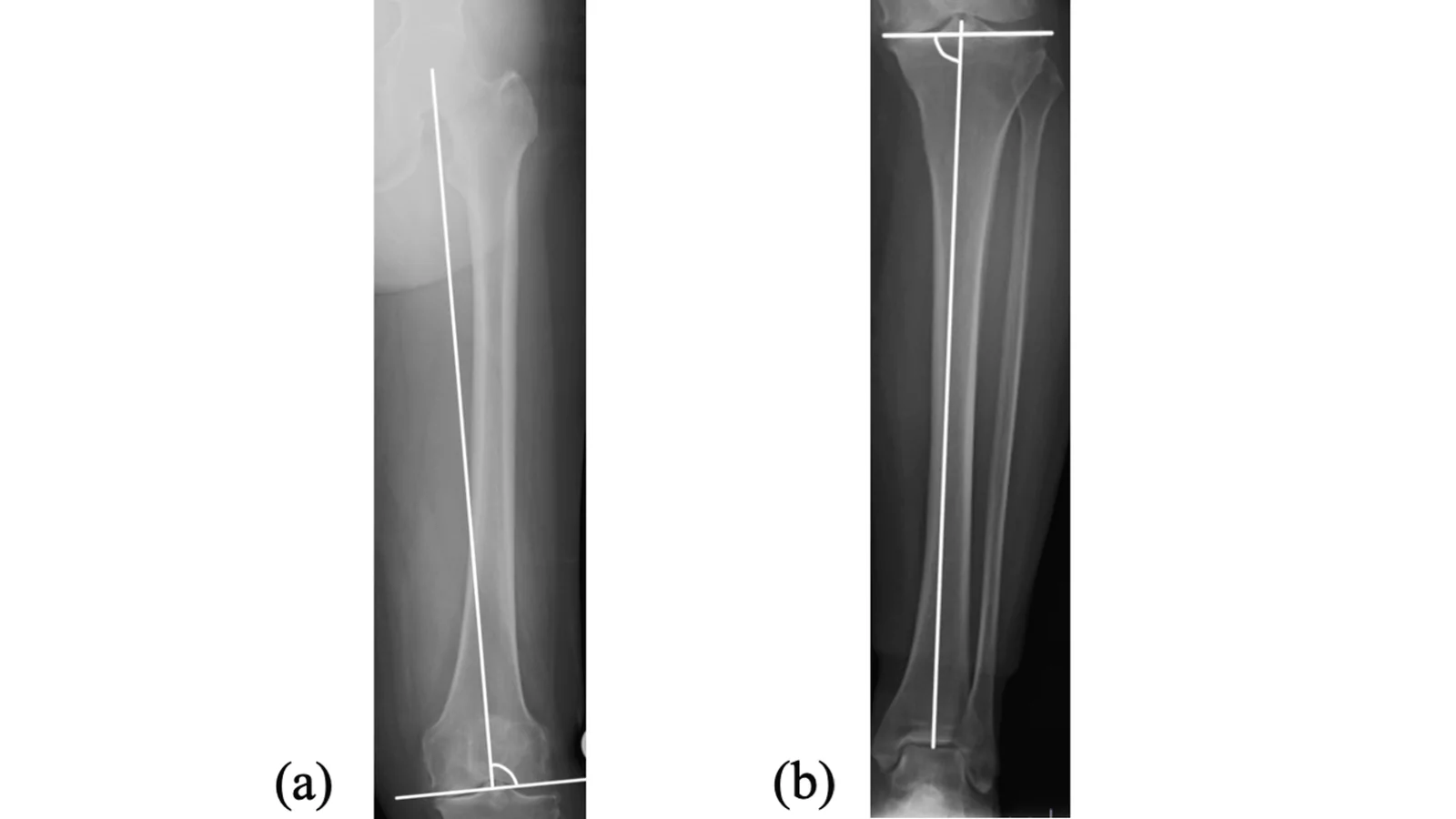
Measurement of the LDFA and MPTA on standing LLRs (a) LDFA: the angle between the femoral mechanical axis and the distal femoral joint line. (b) MPTA: the angle between the tibial mechanical axis and the proximal tibial joint line LDFA: lateral distal femoral angle; MPTA: medial proximal tibial angle; LLR: long-leg radiograph

Standing LLRs were obtained with the patient in a natural standing position, with both lower limbs fully extended and the patellae facing forward. The source-to-image distance was approximately 250 cm. A three-exposure technique was used to obtain a full-length radiograph including the hip, knee, and ankle joints. For standing LLRs, the knee center was defined as the midpoint between the medial and lateral edges of the tibial plateau, excluding osteophytes. The femoral mechanical axis was defined as the line connecting the center of the femoral head to the center of the knee joint, whereas the tibial mechanical axis was defined as the line connecting the center of the knee joint to the center of the ankle joint. The LDFA was defined as the angle between the femoral mechanical axis and the distal femoral joint line (Figure 2a). The MPTA was defined as the angle between the tibial mechanical axis and the proximal tibial joint line (Figure 2b) [1,8].

All measurements for the main analysis were performed by the first author. To evaluate measurement reliability, 10 randomly selected cases were remeasured. Intra-observer reliability was assessed by repeated measurements performed by the first author approximately two months apart, whereas inter-observer reliability was assessed independently by the first author and another orthopedic surgery resident using the same 10 cases. Intraclass correlation coefficients (ICCs) were calculated.

CT

CT scans covering the region from the hip to the ankle joint were performed preoperatively for surgical planning. The scanning parameters were a tube voltage of 120 kV and automatic exposure control with a tube current of 200-400 mA. The CT images were reconstructed with a slice thickness of 2.0 mm for the hip and ankle joints and 0.5 mm for the knee joint. The reconstructed CT data were transferred to the Mako Preoperative Planning Software (Stryker, Mahwah, NJ), where three-dimensional bone models were reconstructed from the CT images, and anatomical landmarks for lower-limb alignment measurements were identified.

**Figure 3:**
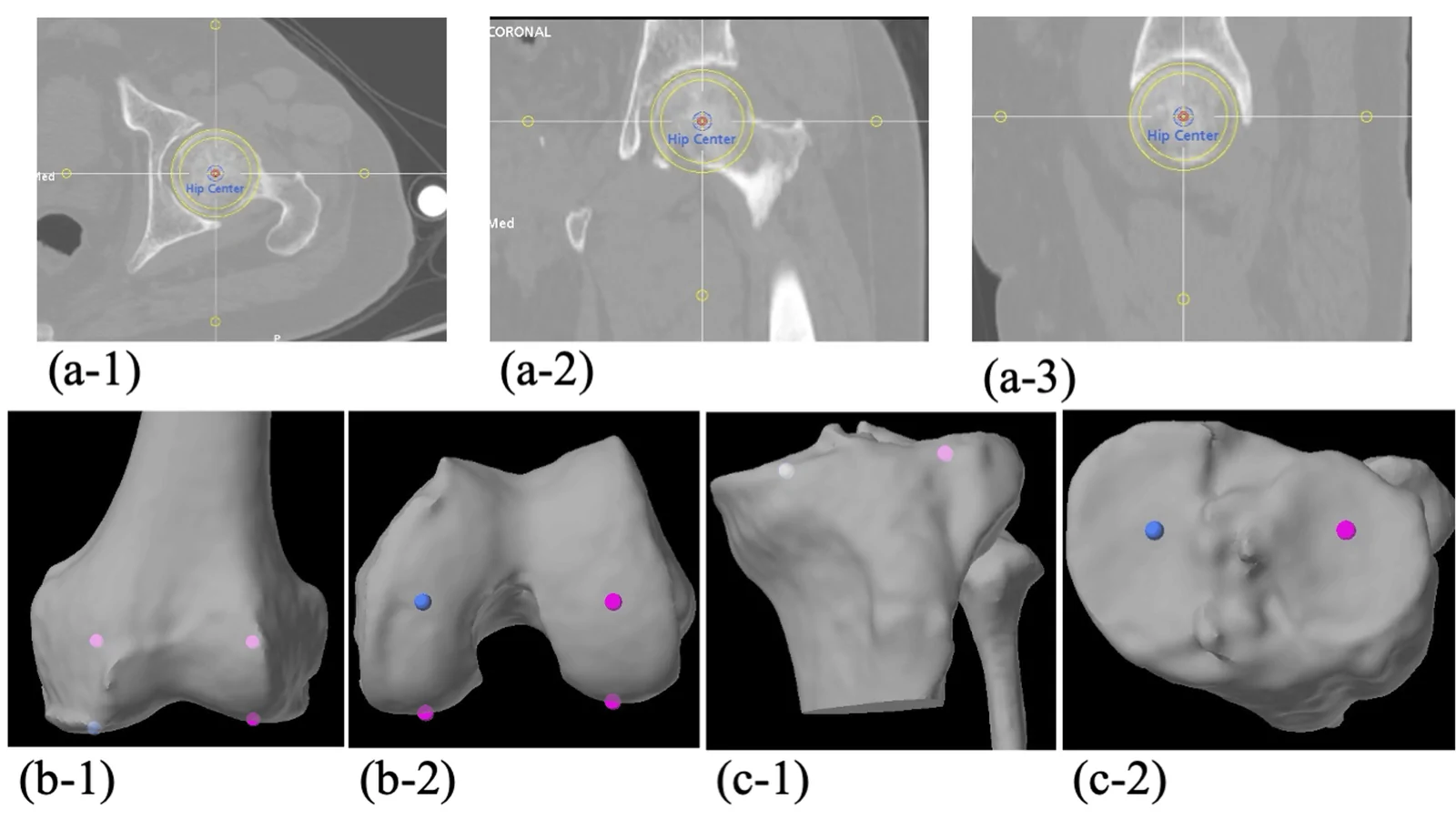
Measurement landmarks on CT images (a-1) Center of the femoral head on the axial view. (a-2) Femoral center on the coronal view. (a-3) Femoral center on the sagittal view. (b-1, 2) Landmarks of the femoral condyles. (c-1,2) Landmarks of the proximal tibial joint surface CT: computed tomography

The knee center was determined using the predefined tibial knee center in the Mako Preoperative Planning Software. The LDFA and MPTA were calculated using the same definitions as those used for the LLR measurements. The center of the femoral head was identified on the axial, coronal, and sagittal CT views (Figure 3a). The distal femoral landmarks were defined as the most distal points of the femoral condyles (Figure 3b). The sagittal landmark of the proximal tibial joint surface was defined as the point located two-thirds of the anteroposterior length of the tibial plateau from its anterior edge (66% point) (Figure 3c) [1,16].

All CT measurements and calculations were performed by an independent product specialist using the standardized measurement protocol implemented in the software. Because this was a retrospective study, the product specialist was blinded to the LLR measurement results and the study hypothesis.

CPAK classification

The MPTA and LDFA measured on LLR and CT were used to calculate the aHKA and JLO using the following equations [8,17]:

$\displaystyle{\displaylines{aHKA = MPTA &minus; LDFA JLO = MPTA + LDFA}}$

Based on the calculated aHKA and JLO values, each knee was classified using the CPAK classification system [8].

Statistical analysis

Continuous variables are presented as mean ± standard deviation (SD). LLR and CT measurements were compared using paired t-tests. Because each knee was evaluated using both LLR and CT, differences between the two imaging modalities in the distribution of CPAK classifications were analyzed using the McNemar-Bowker test to assess changes in classification [18]. All statistical analyses were performed using IBM SPSS Statistics version 23 (IBM Corp., Armonk, NY). A p-value < 0.05 was considered statistically significant.

## Results

Patient characteristics

A total of 90 patients (97 knees) were included in the analysis. The patient characteristics are summarized in Table 1.

**Table 1:** Patient characteristics BMI: body mass index

Variable	Value
Mean age, years (range)	73.4 (49-90)
Sex ratio (male-female)	31:66
Mean BMI, kg/m^2 ^(range)	26.5 (17.0-40.4)
Laterality (right-left)	47:50

Comparison between LLR and CT measurements

Table 2 shows the comparison of lower limb alignment measurements obtained using LLRs and CT, including the mean paired differences (CT - LLR) and their 95% confidence intervals (CIs). There were significant differences between the two imaging modalities in all parameters, namely the MPTA, LDFA, aHKA, and JLO (p < 0.05).

**Table 2:** Comparison of lower limb alignment measurements between LLRs and CT Mean values (± SD) of MPTA, LDFA, aHKA, and JLO measured using LLR and CT are presented, together with the mean paired differences (CT – LLR), 95% CI, and p-values LLR: long-leg radiograph; CT: computed tomography; SD: standard deviation; CI: confidence interval; MPTA: medial proximal tibial angle; LDFA: lateral distal femoral angle; aHKA: arithmetic hip-knee-ankle angle; JLO: joint line obliquity

Variable	LLR, mean ± SD	CT, mean ± SD	Mean paired difference (95% CI)	P-value
MPTA	85.3 ± 2.36	83.8 ± 2.23	-1.5 (-1.7 to -1.3)	< 0.01
LDFA	88.6 ± 2.43	87.5 ± 2.18	-1.1 (-1.3 to -0.8)	< 0.01
aHKA	-3.0 ± 4.0	-3.0 ± 3.3	-0.4 (-0.8 to -0.1)	0.02
JLO	173.9 ± 3.1	171.4 ± 2.92	-2.6 (-2.9 to -2.3)	< 0.01

**Figure 4:**
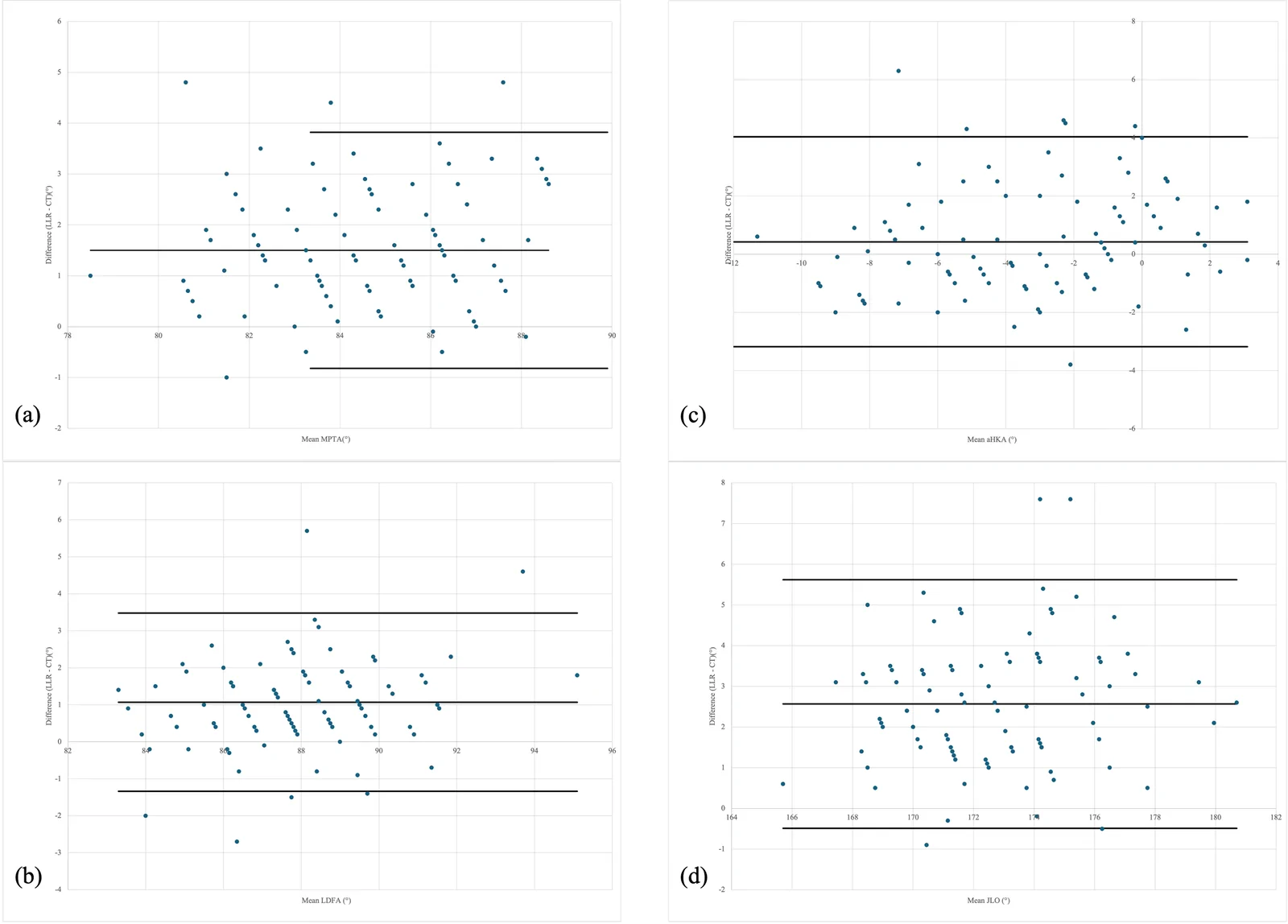
Bland-Altman plots comparing standing LLRs and CT measurements (a) MPTA, (b) LDFA, (c) aHKA, and (d) JLO. The central horizontal line indicates the mean difference (bias), and the upper and lower horizontal lines indicate the 95% limits of agreement (bias ± 1.96 SD) LLRs: long-leg radiographs; CT: computed tomography; MPTA: medial proximal tibial angle; LDFA: lateral distal femoral angle; aHKA: arithmetic hip-knee-ankle angle; JLO: joint line obliquity; SD: standard deviation

Bland-Altman analysis was performed to assess the agreement between standing LLRs and CT measurements (Figure 4). The mean differences (biases) were 1.50° for MPTA, 1.07° for LDFA, 0.43° for aHKA, and 2.56° for JLO. The corresponding 95% limits of agreement were −0.82° to 3.82° for MPTA, −1.34° to 3.48° for LDFA, −3.18° to 4.04° for aHKA, and −0.49° to 5.62° for JLO.

The intra-observer reliability was good to excellent, with ICC values of 0.88 for LDFA, 0.97 for MPTA, 0.95 for aHKA, and 0.94 for JLO. The inter-observer reliability was acceptable to good, with ICC values of 0.75 for LDFA, 0.88 for MPTA, 0.88 for aHKA, and 0.80 for JLO, indicating good reproducibility of the measurements.

Table 3 presents the comparison of CPAK classification distributions between LLRs and CT assessed using the McNemar-Bowker test. There was no significant difference between the two imaging modalities in the CPAK classification distribution, and the overall distribution patterns were similar.

**Table 3:** Distribution of CPAK classification types based on LLR and CT measurements Differences in the distribution between the two imaging modalities were analyzed using the McNemar-Bowker test CPAK: coronal plane alignment of the knee; LLRs: long-leg radiographs; CT: computed tomography; df: degrees of freedom

CPAK Type	LLR, % (n)	CT, % (n)	X^2^ (df)	P-value
Apex distal JLO				
CPAK Type Ⅰ	44.3 (43)	59.8 (58)	3.9 (2)	0.142
CPAK Type Ⅱ	30.9 (30)	32.0 (31)		
CPAK Type Ⅲ	3.1 (3)	4.1 (4)		
Neutral JLO				
CPAK Type Ⅳ	13.4 (13)	4.1 (4)	N/A	N/A
CPAK Type Ⅴ	8.3 (8)	0		
CPAK type Ⅵ	0	0		
Apex proximal JLO				
CPAK Type Ⅶ	0	0	N/A	N/A
CPAK Type Ⅷ	0	0		
CPAK Type Ⅸ	0	0		

CPAK classification changed in 29 of 97 knees (29.9%) between standing LLRs and CT, whereas 68 knees (70.1%) remained in the same classification. The transitions between CPAK classifications are summarized in Table 4. Most classification changes occurred from Types IV and V to Types I and II.

**Table 4:** Transition of CPAK classifications between standing LLRs and CT Values represent the number of knees. Rows indicate CPAK classifications determined using standing LLRs, and columns indicate CPAK classifications determined using CT. Diagonal cells represent knees with unchanged CPAK classifications, whereas off-diagonal cells represent knees with changes in classification between the two imaging modalities CPAK: coronal plane alignment of the knee; LLR: long-leg radiograph; CT: computed tomography

LLR ＼CT	Type Ⅰ	Type Ⅱ	Type Ⅲ	Type Ⅳ	Type Ⅴ
Type Ⅰ	42	1	0	0	0
Type Ⅱ	6	21	2	0	0
Type Ⅲ	0	1	2	0	0
Type Ⅳ	7	4	0	3	0
Type Ⅴ	3	4	0	1	0

Among the 29 knees in which the CPAK classification changed, the change was attributable to crossing the JLO cutoff in 13 knees (44.8%), the aHKA cutoff in 10 knees (34.5%), and both cutoffs in six knees (20.7%).

**Figure 5:**
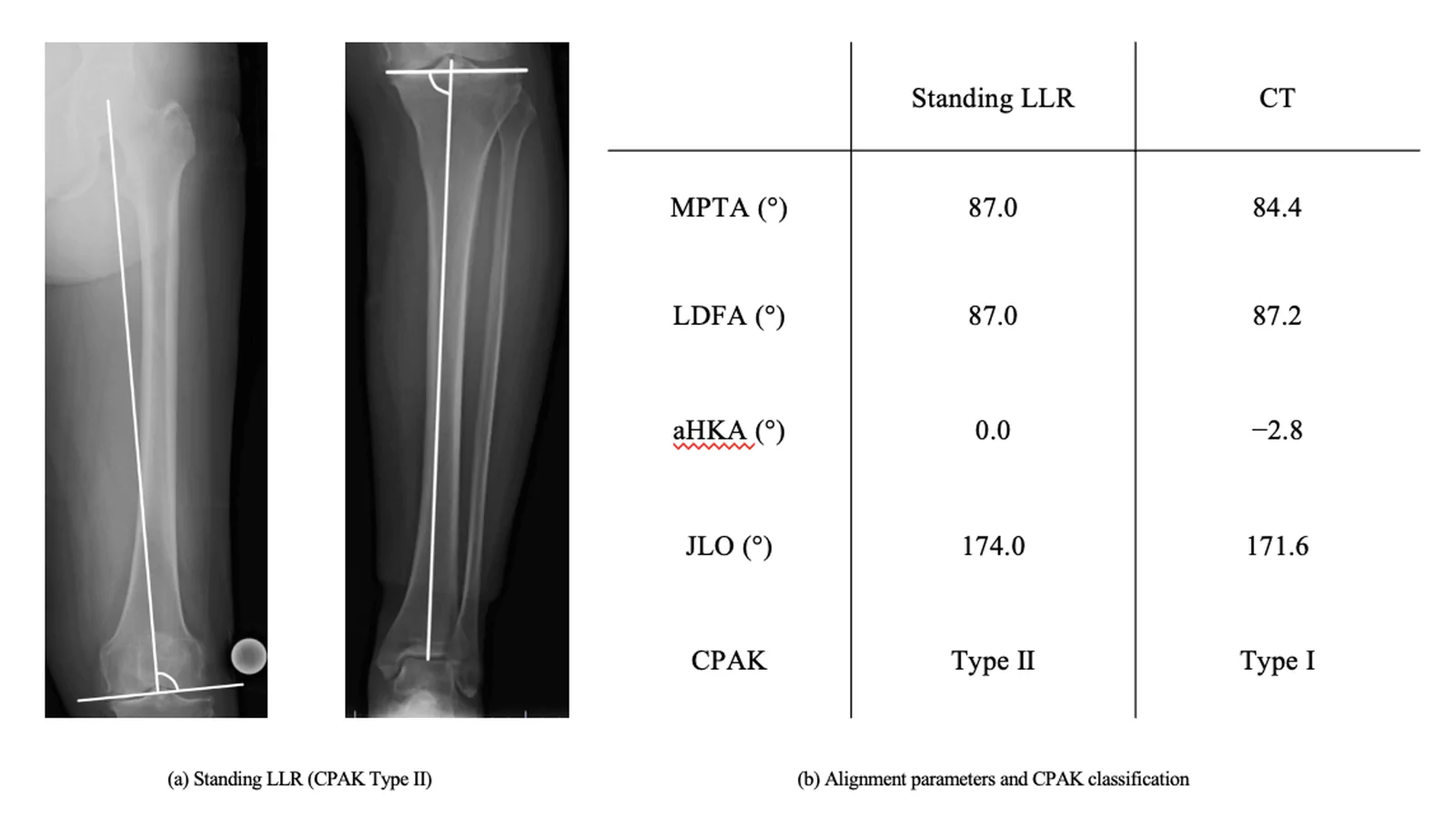
Representative case showing a change in CPAK classification between standing LLRs and CT (a) Standing LLR demonstrating the measurements used for lower limb alignment assessment. (b) Comparison of alignment parameters and CPAK classification between standing LLR and CT. In this representative case, the CPAK classification changed from Type II on standing LLR to Type I on CT CPAK: coronal plane alignment of the knee; LLRs: long-leg radiographs; CT: computed tomography

**Figure 6:**
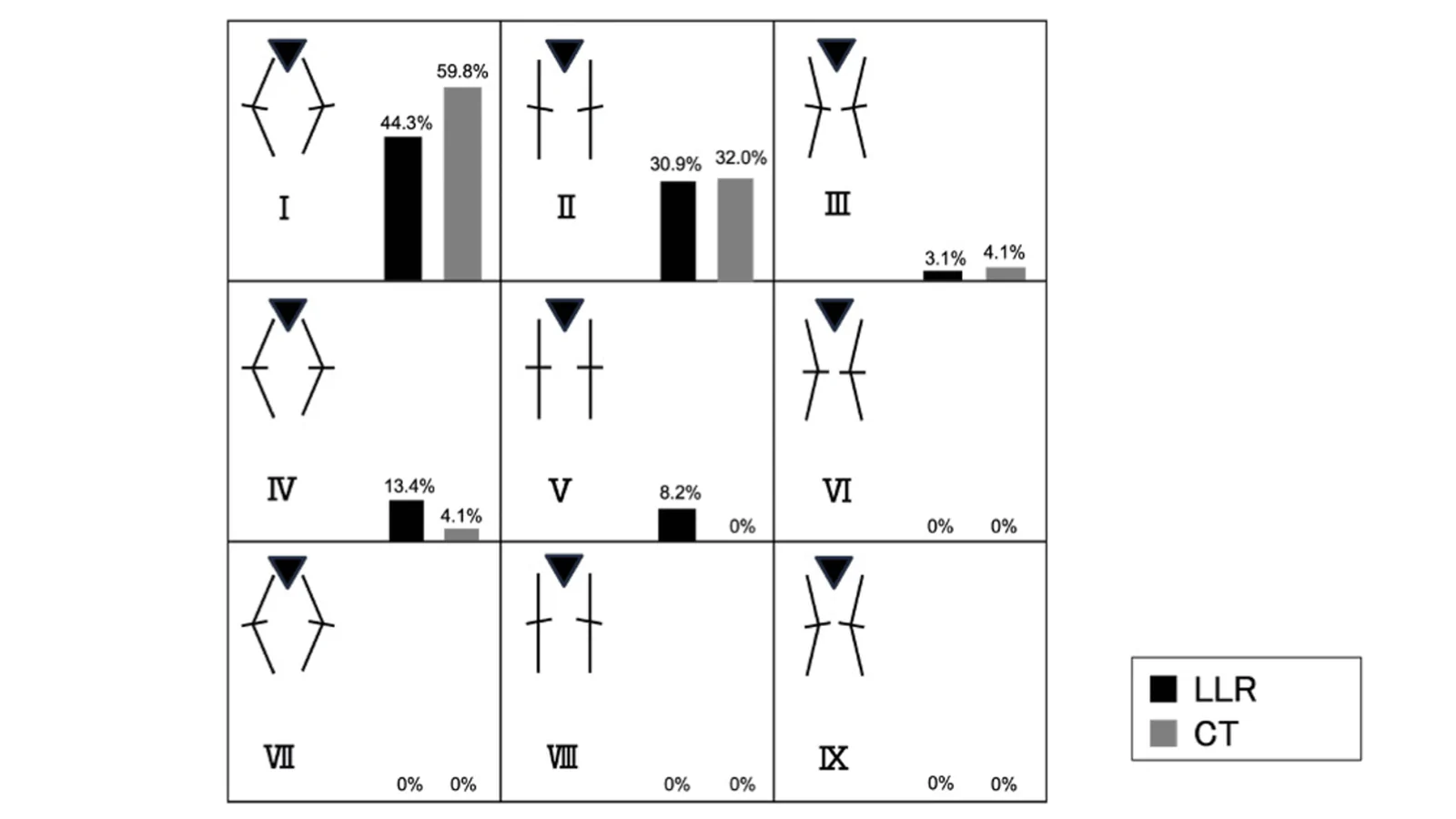
Distribution of CPAK classification types determined on standing LLRs and CT Each bar represents the proportion of knees classified into each CPAK type based on LLR and CT measurements CPAK: coronal plane alignment of the knee; LLRs: long-leg radiographs; CT: computed tomography

A representative case showing a change in CPAK classification from Type II on standing LLR to Type I on CT is presented in Figure 5. The proportions of CPAK classification types determined by LLRs and CT are shown in Figure 6. Compared with LLRs, CT showed an increased proportion of knees classified as CPAK Types I-III and a decreased proportion of knees classified as Types IV and V.

## Discussion

The main findings of this study were that standing LLRs significantly underestimated varus lower limb alignment and JLO compared with CT in a Japanese population. In addition, although the distribution of CPAK classification types did not significantly differ between imaging modalities, a trend toward differences in distribution was observed with CT-based evaluation. These findings are consistent with previous studies conducted in Western populations [1]. LLR measurements are known to be influenced by limb position, weight-bearing conditions, and rotational alignment during imaging, whereas CT is less affected by limb position and weight-bearing conditions and enables assessment based on bony morphology [1,2,3,8]. In the present study, the aHKA and JLO measured on CT indicated greater varus alignment and greater JLO than the measurements on LLRs, suggesting a similar tendency in the Japanese population.

Previous studies have reported that Japanese patients exhibit characteristic tibial and femoral morphologies that influence lower limb alignment [9,10]. The greater varus alignment and JLO observed on CT in the present study, compared with values reported in Western populations [1], may reflect these morphological characteristics. In other words, LLRs, which are susceptible to imaging conditions, may fail to accurately represent constitutional alignment determined by bony morphology in some patients in Asian populations.

In contrast to the differences in varus alignment and JLO, there were no significant differences in the distribution of CPAK classifications assessed using LLRs versus CT in the present study. This finding differs from previous studies conducted in Western populations and may represent a characteristic feature of the Japanese population. Previous reports have shown that CPAK type I is the most prevalent phenotype among Japanese patients with knee osteoarthritis [6]. Therefore, even when CT evaluation results in greater varus alignment and JLO, changes in CPAK classification may affect only a small number of knees, which may explain the absence of a significant difference in the overall distribution of CPAK types. As discussed by Tarassoli et al. [1], differences in the definition of the proximal tibial joint line between standing LLR and CT may also contribute to differences in MPTA measurements. This effect may be more pronounced in knees with advanced varus deformity and may influence CPAK classification in cases near the classification boundaries.

The present results suggest that differences in evaluation methods may not substantially alter CPAK classification types in Japanese patients, but may instead influence cases that lie near the boundaries between CPAK types. Bland-Altman analysis demonstrated systematic differences between standing LLR and CT measurements across all alignment parameters. Although the mean biases were relatively small, the 95% limits of agreement indicated that differences of several degrees may occur between the two modalities in individual cases. These results further support the observation that differences between imaging modalities may particularly influence knees with measurements close to CPAK classification boundaries. The representative case shown in Figure 5 illustrates this finding, demonstrating that relatively small differences in alignment measurements may alter the CPAK classification when values are close to the classification boundaries.

The CPAK classification has been proposed as a useful framework for preoperative planning in TKA [8]. Accurate preoperative angular assessment is essential because kinematic alignment aims to restore the patient’s constitutional alignment [8,19,20]. Based on the present findings, CT evaluation in Japanese patients undergoing TKA may be useful for preoperative alignment assessment because it demonstrated greater varus alignment and JLO than LLR.

Limitations

This study has several limitations. First, this was a retrospective study with a limited sample size of 90 patients (97 knees). Therefore, the sample size may have been too small to detect a statistically significant difference in the distribution of CPAK classifications. Second, this study focused on alignment assessment based on bony morphology and did not evaluate the influence of ligament balance or soft tissue conditions. These factors may affect actual knee function and postoperative outcomes and should be investigated in future studies.

Third, LLRs were obtained under weight-bearing conditions, whereas CT was performed in the supine position. Differences between these imaging modalities in limb position and loading conditions may have influenced the measurement differences observed in this study. In addition, supine LLRs were not routinely obtained at our institution and were therefore unavailable for analysis in this retrospective study, while standing CT was not available during the study period. Therefore, the effects of imaging modality could not be clearly distinguished from those of weight-bearing conditions and patient positioning. Future studies comparing supine LLRs with CT and incorporating standing CT may help clarify the respective effects of imaging modality, loading conditions, and patient positioning on lower-limb alignment measurements. Fourth, seven patients contributed bilateral knees to the analysis. Because the left and right knees from the same patient are not completely independent observations, some degree of statistical dependence may have been present. However, bilateral cases accounted for only a small proportion of the study cohort, and their effect on the overall results was considered limited.

Fifth, intra- and inter-observer reliability was not independently evaluated for CT measurements. Although CT measurements were performed using the standardized workflow implemented in the Mako Preoperative Planning Software by a product specialist, the reproducibility of CT-based measurements was not specifically assessed in this study. Future studies should evaluate the reliability of CT measurements. Finally, this was a single-center study conducted in a Japanese population. Therefore, caution should be exercised when generalizing these findings to other ethnic groups or populations with different clinical backgrounds.

## Conclusions

In a Japanese population, standing LLRs underestimated the degree of varus lower limb alignment and JLO compared with CT. Although the overall distribution of CPAK classifications showed similar trends between the two imaging modalities, no statistically significant difference was observed. These findings suggest that CT demonstrates greater varus alignment and JLO than LLRs in Japanese patients and may be particularly useful for evaluating knees that lie near the boundaries between CPAK classification types.
